# Metabolic Engineering of *Escherichia coli* for Methyl Parathion Degradation

**DOI:** 10.3389/fmicb.2022.679126

**Published:** 2022-02-11

**Authors:** Jing Xu, Bo Wang, Ming-Qing Wang, Jian-Jie Gao, Zhen-Jun Li, Yong-Sheng Tian, Ri-He Peng, Quan-Hong Yao

**Affiliations:** Shanghai Key Laboratory of Agricultural Genetics and Breeding, Biotechnology Research Institute, Shanghai Academy of Agricultural Sciences, Shanghai, China

**Keywords:** methyl parathion, multigene metabolic engineering, methyl-parathion degradation, genetically engineered bacteria, synthetic biology

## Abstract

Organophosphate compounds are widely used in pesticides to control weeds, crop diseases, and insect pests. Unfortunately, these synthetic compounds are hazardous and toxic to all types of living organisms. In the present work, *Escherichia coli* was bioengineered to achieve methyl parathion (MP) degradation via the introduction of six synthetic genes, namely, *opdS*, *pnpAS*, *pnpBS*, *pnpCS*, *pnpDS*, and *pnpES*, to obtain a new transformant, BL-MP. MP and its subsequent decomposition intermediates were completely degraded by this transformant to enter the metabolites of multiple anabolic pathways. The MP-degraded strain created in this study may be a promising candidate for the bioremediation of MP and potential toxic intermediates.

## Introduction

Methyl parathion (MP; *O*,*O*-dimethyl *O*-4 nitrophenyl phosphorothioate) is an organophosphorus pesticide that is widely used in agriculture to protect agricultural crops from insect pests and, thus, increase food production (Bhatt et al., 2021b). However, the increasing use of pesticides in agriculture poses potential hazards to aquatic organisms, for example, by interfering with normal health, developmental, and reproductive processes (Martin-Reina et al., 2017). Moreover, the accumulation of MP also contaminates dairy products (Patnaik and Padhy, 2016). MP could be slowly hydrolyzed (*t*_1/2_ = 68 days at pH 5, *t*_1/2_ = 40 days at pH 7, and *t*_1/2_ = 33 days at pH 9) in buffer solutions and slowly photodegraded (*t*_1/2_ = 61 days) on soil surfaces (US Environmental Protection Agency [USEPA], 1999). The high environmental persistence of MP residues in food and water could impose great threats (Huang et al., 2010). The main neurotoxic effects of MP are associated with inhibition of acetyl-cholinesterase (AChE) activity and prevention of acetylcholine (ACh) hydrolysis. Excessive ACh levels can cause overstimulation of the cholinergic system and even death within minutes (Garcia et al., 2003). Delayed neurotoxicity and polyneuropathy have also been described as the effects of MP toxicity and its residual concentration (Hsieh et al., 2001). Therefore, MP has been classified as extremely hazardous and listed in the HazDat database of “Chemicals Detected in Surface and/or Groundwater” at the National Priorities List (NPL) sites (WHO, 2004).

Traditionally, physical and chemical methods, including incineration and chemical hydrolysis (De La Peña Mattozzi et al., 2006), have been developed to eliminate organophosphates. However, the available methods usually involve sophisticated processes, expensive equipment, and high energy requirements (Ma et al., 2014).

Additionally, methyl paraoxon, the main oxidation product produced by oxidative desulphurization, has been considered to be approximately three times more toxic than MP (Straus et al., 2000). On the other hand, 4-nitrophenol (PNP), the major hydrolysis product, also presents acute toxicity and mutagenic potential. These compounds pose potential threats to the environment and public health (Suja et al., 2012).

Bioremediation based on microbial metabolism has been shown to be more effective and eco-friendly than physical and chemical methods in the detoxification of organophosphates (Cycon and Piotrowska-Seget, 2016). Many microorganisms can hydrolyze MP by using organophosphate acid anhydrases (e.g., Opd) for detoxification. This hydrolase has been found in MP-degrading bacteria, such as *Pseudomonas*, *Stenotrophomonas*, *Plesiomonas*, *Achromobacter xylosoxidans*, *Ochrobactrum tritici*, *Brucella melitensis*, and *Burkholderia* (Cui et al., 2001; Zhang et al., 2005, 2006; Qiu et al., 2006; Shen et al., 2010; Ramu and Seetharaman, 2014; Deng et al., 2015). The initial hydrolysis of MP by Opd to PNP reduces the toxicity of the metabolite by 120-fold (Munnecke, 1979). However, although it is much less toxic than MP, PNP is still classified as a priority and persistent toxic contaminant (Leung et al., 2005; Kulkarni and Chaudhari, 2006). Many PNP-degrading bacteria have been isolated, and their degradation characteristics have been extensively studied (Ju and Parales, 2010). PNP can be aerobically degraded by two different pathways: meta-cleavage and ortho cleavage (Kitagawa et al., 2004). The intermediates of these two pathways are BT and HQ. The final product of both pathways, β-ketoadipate, can subsequently enter metabolites through a variety of anabolic pathways, including the tricarboxylic acid (TCA) cycle and fatty acid biosynthesis (Wells and Ragauskas, 2012).

**FIGURE 1:**
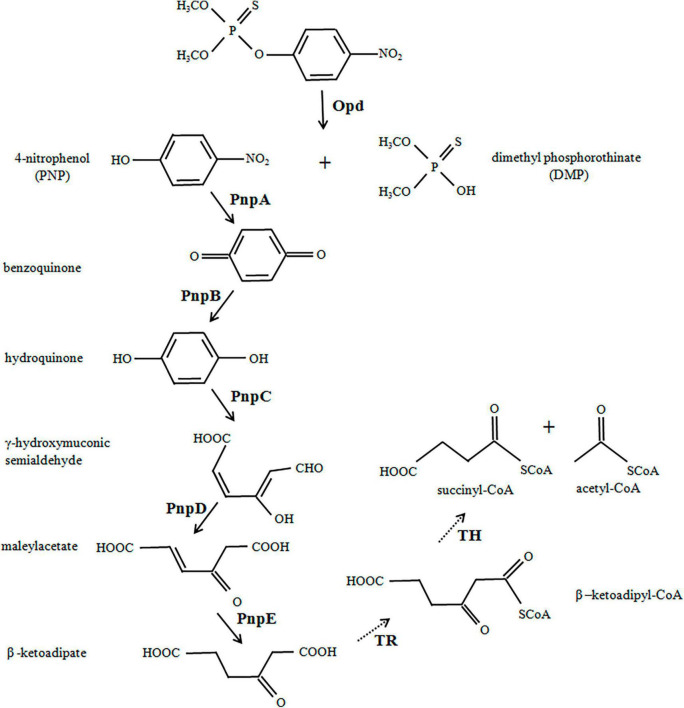
Engineered biodegradation pathway for methyl parathion. Organophosphate hydrolase (Opd) from *Flavobacterium* sp. ATCC 27551 initially hydrolyzed the methyl parathion (MP) into two major intermediate products, 4-nitrophenol (PNP) and dimethylphosphate (DMP). PNP was further converted to β-ketoadipate under the catalysis of five enzymes (encoded by *pnpA*–*pnpE*) from *Pseudomonas putida*. The solid arrows show enzyme actions as designed, and the dotted arrows show spontaneous reactions in bacteria.

For the first time, an *Escherichia coli* strain was successfully constructed in the present work to directionally degrade toxic MP into the metabolites of multiple anabolic pathways by using the method of synthetic biology (Figure 1). Six genes, *opd* (organophosphate acid hydrase) from *Flavobacterium* sp. ATCC 27551 and *pnpA*–*pnpE* (genes for degradation of PNP to β-ketoadipate) from *Pseudomonas putida* were chemically synthesized. The relevant codons were also modified and optimally designed for the engineered strain. The open reading frames of the six genes were seamlessly connected to the T7 promoter and terminator to construct expression cassettes, which were then inserted into the pCAMBIA1301 vector to generate the recombinant MP-degrading *E. coli* strain. Our results confirmed that the complete degradation of MP by *E. coli* could be achieved by introducing six degradation genes into the designed biodegradation pathway of the organophosphate. The MP-degrading strain created in this study may be a promising candidate for the bioremediation of MP and other potential toxic intermediates. The method adopted in the study may also be applied to transform or create strains that can adapt to different environments or break down other pollutants.

## Materials and Methods

### Reagents and Culture Conditions

MP (AR, 98%) was purchased from Molbase (Shanghai, China)^1^. PNP (AR, 98%) was purchased from Aladdin (Shanghai, China)^2^. β-Ketoadipate (AR, 98%) was purchased from Finetech Industry Limited (Wuhan, China). All other chemicals were purchased from Sangon Biotech Co., Ltd. (Shanghai, China). Primers were synthesized from Sangon Biotech Co., Ltd. KOD DNA polymerase was purchased from TOYOBO Co., Ltd. (Osaka, Japan). Restriction enzymes were purchased from Takara Biomedical Technology Co., Ltd. (Beijing, China).

*Escherichia coli* strains BL21-AI were grown in LB medium at 37°C. Media were supplemented with kanamycin (30 μg/ml) when required.

### Vector Construction

The pGEM-T easy plasmid (Promega, Beijing, China) and *E. coli* strain DH5α (Invitrogen, Carlsbad, CA, United States) were used for gene cloning and vector construction. The pCAMBIA1301 (Novagen, Madison, WI, United States) and *E. coli* strain BL221-AI (Invitrogen, Carlsbad, CA, United States) were employed to express the artificial gene cluster in this study. *opd* gene (GenBank: AJ421424) for MP hydrolysis and five genes *pnpA*–*pnpE* (GenBank: FJ376608.2) for PNP degradation were chemically synthesized using PCR-based two-step DNA synthesis (PTDS) (Xiong et al., 2004, 2006). The codons of the six genes were modified and optimally designed for *E. coli*^3^. Synthetic *opd*, *pnpA*, *pnpB*, *pnpC*, *pnpD*, and *pnpE* were named *opdS*, *pnpAS*, *pnpBS*, *pnpCS*, *pnpDS*, and *pnpES*, respectively (GenBank: MZ393850, MZ393851, MZ393852, MZ393853, and MZ393854, respectively).

**FIGURE 2:**
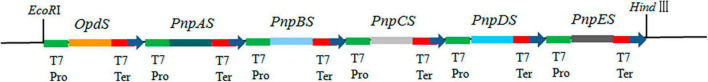
Schematic diagram of construction O*pdS*-*PnpES*. *OpdS* (encoded organophosphate hydrase), *PnpAS* (encoded *p*-nitrophenol 4-monooxygenase), *PnpBS* (encoded *p*-benzoquinone reductase), *PnpCS* (encoded hydroquinone 1,2-dioxygenase), *PnpDS* (encoded γ-hydroxymuconic semialdehyde dehydrogenase), and *PnpES* (encoded maleylacetate reductase). T7 Pro, T7 promoter; T7 Ter, T7 terminator.

The gene expression cassette was then constructed by connecting each ORF between T7 promoter and terminator and putting them in order using the polyacrylamide gel electrophoresis (PAGE)-mediated overlap extension PCR method (Peng et al., 2006). The gene expression cassettes of *opdS*, *pnpAS*, *pnpBS*, *pnpCS*, *pnpDS*, and *pnpES* were designated as T7*opdS*–T7*pnpAS*–T7*pnpBS*–T7*pnpCS*–T7*pnpDS*–T7*pnpES*, abbreviated as T7*opdS*–T7*pnpS*. Meanwhile, the expression cassette was added with *Eco*RI and *Hin*dIII restriction sites to the 5′ and 3′ end of the expression cassette (Figure 2). And the expression cassette was subsequently inserted into the pCAMBIA1301 vector. Finally, the six-gene construction was obtained and introduced into the host *E. coli* strain BL21-AI. The transformants were named BL-MP.

### Growth Condition

The transformants BL-MP were cultivated in M9 medium (Na_2_HPO_4_⋅7H_2_O, 12.8 g/L; KH_2_PO_4_, 3 g/L; NH_4_Cl, 1 g/L; and NaCl, 0.5 g/L), supplemented with casamino acids at 1 g/L, glycerin (replace glucose) at 10 g/L, and thiamine hydrochloride at 10 ppm. Additionally, inducers isopropyl-β-D-thiogalactoside (IPTG) at 1 mM, arabinose at 2 g/L, and antibiotics (kanamycin sulfate at 30 μg/ml) were added to the medium simultaneously.

The bacteria were cultured at 37°C and shaking rate of 160 rpm for 24 h. Cells were harvested by centrifugation (4°C, 7,800 *g*, 8 min) and washed three times with M9. Cells were inoculated at OD_600_ = 0.5 in 50 ml of M9 medium containing antibiotics and inducers for subsequent use.

### Gene Expression Analysis

The transformants (BL-MP) carrying all the six genes were used for RNA extraction. After 8 h of induction, total RNA from the BL-MP was extracted using TRIzol reagent (Invitrogen, Waltham, MA, United States) according to the manufacturer’s instruction. Synthesis of cDNA was using cDNA Synthesis superMix (TransGen Biotech Co., Ltd., Beijing, China) according to the manufacturer’s instruction. The quantitative reverse transcriptase PCR (qRT-PCR) of six genes was performed according to the method of Wang et al. (2019); the 16S rRNA gene was used as an internal control. The gene expression is relative to 16S rRNA expression. The primer sequences for each gene used are listed in Supplementary Table 1.

### Methyl Parathion Biodegradation

For detecting the biodegradation capability of the transformed strains, 1 mM of MP or 1 mM of PNP were added to the prepared bacterial suspension containing inducers. Under the same conditions, the strains transformed with empty vector were used as the control group, and M9 medium containing inducers and without strains was used as the blank group.

### Analysis of Main Metabolites of Methyl Parathion Degradation

Cell densities were analyzed with optical densities at 600 nm (OD_600_).

The growth of BL-MP in M9 medium with 1 mM of MP was monitored over a 3-day period. Two methods were used to analyze the concentration of MP and its main hydrolysis products, PNP, hydroquinone (HQ), and β-ketoadipate.

#### High-Performance Liquid Chromatography

High-performance liquid chromatography (HPLC) was performed using an Agilent 1100 HPLC system (Agilent Technologies, Santa Clara, CA, United States) equipped with Athena 5 μm of C18 column (4.6 × 150 mm, CNW) (Agilent) and ultraviolet spectrophotometric detector (Agilent 1100 VWD). Twenty microliters of the sample was tested. The mobile phase consisted of 50% acetonitrile and 50% ultrapure water at a flow rate of 1 ml min^–1^ for MP, 50% methanol and 50% ultrapure water at 0.5 ml min^–1^ for PNP, and 30% methanol and 70% ultrapure water at 0.5 ml min^–1^ for HQ. The detection wavelength for these three chemicals was 278, 318, and 270 nm.

#### Gas Chromatography–Mass Spectrometry

β-Ketoadipate produced in the sample was detected by gas chromatography–mass spectrometry (GC-MS) after derivatization, using the method of Okamura-Abe et al. (2016) and Wang et al. (2019) with minor modifications. GC-MS/MS, 7890B-7000C (Agilent), was equipped with an HP-5 MS column (30 m × 0.25 mm × 0.25 μm, Agilent). The heating procedure of column oven temperature was as follows: from 100 to 160°C at a rate of 40°C/min, from 160 to 250°C at a rate of 10°C/min, and from 250 to 300°C at a rate of 20°C/min. The ion source temperature was 230°C, and mass spectra from *m*/*z* 50 to 400 were recorded under electron ionization at 70 eV.

Error bars in all figures represent the SD of the mean unless otherwise noted. Metabolite concentrations were measured from three biologically independent samples.

## Results and Discussion

The Green Revolution has remarkably increased total grain production and promoted the development of agriculture, but the increased use of agricultural chemicals (including pesticides) has also caused serious environmental problems. Organophosphates, the second major group of pesticides, are widely used for agricultural pest control; however, these substances are hazardous and/or toxic to all types of living organisms (Mulla et al., 2020). Some bacteria living in various environments are unique in that they can break down and use different organophosphate pesticides for growth (Singh and Walker, 2006; Liu et al., 2007; Siripattanakul-Ratpukdi et al., 2014; Nair et al., 2015; Rayu et al., 2017). However, microorganisms that are suitable for bioremediation and biodegradation are scarce because of (i) the long periods of natural evolution and (ii) various abiotic and biotic factors that affect their effectiveness, such as the type of contaminant, environmental constraint, and indigenous microbial ecology. Synthetic biology is a powerful tool to offer the possibility of overcoming these difficulties within a relatively short time and create engineered strains with enhanced degradative abilities for bioremediation by the assembly of pathways using enzymes from multiple organisms (Copley, 2009). The main purpose of this study is to achieve the complete biodegradation of MP, a common organophosphate, through genetic engineering.

### Construction of a Synthetic Operon for Methyl Parathion and 4-Nitrophenol Hydrolysis

The first step in this work is to design, construct, and optimize an operon to hydrolyze MP and PNP. The degradation scheme is shown in Figure 1. In short, Opd hydrolyzes MP to PNP, which is then converted to β-ketoadipate under the action of various enzymes. The metabolite enters the TCA cycle through the acetyl-CoA and succinyl-CoA encoded by the host. All six genes for MP degradation, namely, *opd*, *pnpA*, *pnpB*, *pnpC*, *pnpD*, and *pnpE*, were obtained by chemical synthesis. All codons were designed and optimized to enhance gene expression.

In recent years, our laboratory has successfully constructed bacterial multigene engineering strains (Wang et al., 2019, 2021). Most metabolic engineering studies on prokaryotes are based on the modification of operon genes, and an essential requirement of multigene engineering is the induction of multiple genes to express simultaneously in a coordinated manner within a heterologous microorganism background (Wu et al., 2016). In plants, the use of the same promoter for each gene can ensure the coordinated expression of multiple genes (Zhu et al., 2008; Tian et al., 2020). In transgenic microorganisms, the same operation for multiple transgenes controlled by the same promoter can also achieve coordinated and stable expression (Wang et al., 2019, 2021). Here, the T7 promoter, which is known as one of the strongest expression systems for expressing exogenous genes and recombinant proteins in *E. coli* (Landick, 2004), was selected to control the expression of exogenous genes. The genetic sequencing of the constructed synthetic operon was designed as follows: T7*opdS*–T7*pnpAS*–T7*pnpBS*–T7*pnpCS*–T7*pnpDS*–T7*pnpES* (Figure 2).

### Genetic Transformation of *Escherichia coli*

**FIGURE 3:**
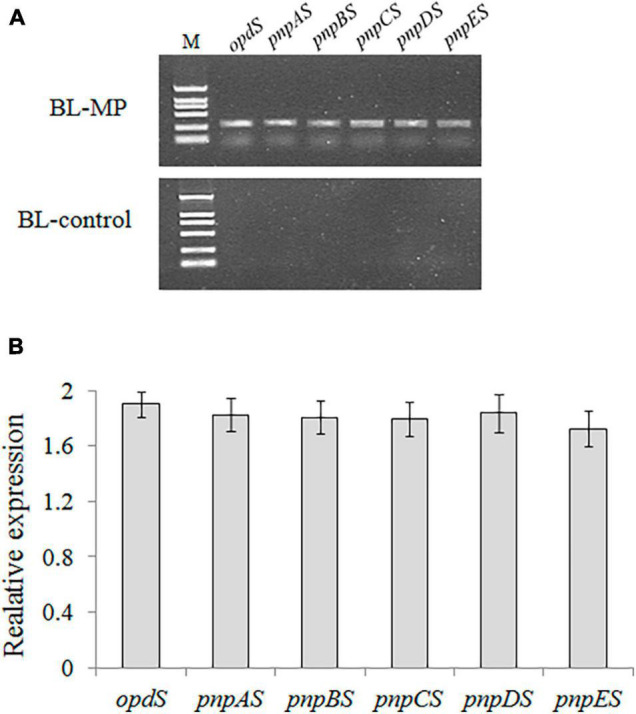
Cloning and expression analyses of all six synthetic genes in the transformant strain BL-MP. **(A)** PCR analysis was performed using the plasmid extracted from BL-MP or BL-control as template. **(B)** The qRT-PCR analysis was performed using cDNA of BL-MP or BL-control as template. The mRNAs extracted from BL-MP or BL-control were used to synthesize the corresponding cDNAs. The gene expression is relative to 16S rRNA expression.

The recombinant vector containing the six genes in the designed MP biodegradation pathway was transformed into the host strain, *E. coli* BL21-AI. PCR confirmed the successful construction of the expression vector (Figure 3A). The transcriptional expressions of the six genes were analyzed using RT-PCR, and the results showed that all six genes are expressed in *E. coli* (Figure 3B). It is thought that the vector containing multiple genes becomes more cumbersome and unstable as the number of genes transferred increases (Zorrilla-López et al., 2013). In this study, gene clusters for MP degradation were artificially constructed using a monocistronic transcriptional model. The results of RT-PCR demonstrated that the six genes designed for the bioconversion of MP to β-ketoadipate were successfully expressed in *E. coli*.

### Analysis of Methyl Parathion Hydrolysis

MP measuring 1 mM was added to the prepared bacterial suspension containing inducers to detect the biodegradation ability of the transformed strains. MP and its hydrolysis products, including PNP, HQ, and β-ketoadipate, were then detected.

**FIGURE 4:**
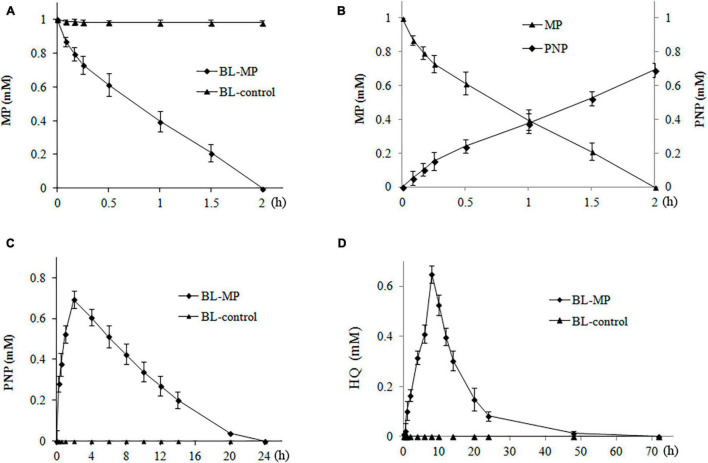
Degradation of 1 mM of methyl parathion (MP). MP was converted to the intermediates 4-nitrophenol (PNP) and hydroquinone (HQ) during the reaction. Values are mean ± SD of three replicates. **(A)** MP concentration at different times. **(B)** The concentration of MP and PNP at different times. **(C)** PNP concentration at different times. **(D)** HQ concentration at different times.

When 1 mM of MP was added to the liquid medium, 13% of MP was degraded within the first 5 min by the transformant BL-MP. Complete degradation of MP was achieved within 2 h (Figure 4A). These results demonstrate that BL-MP has a strong MP-degrading ability.

The concentration of PNP, the first hydrolysis product of MP, increased rapidly within the first 2 h of incubation and peaked at 2 h, which is inversely proportional to the degradation of MP (Figure 4B). Thereafter, the concentrations of PNP continued to decrease between 2 and 20 h of incubation. After 20 h, 96% of PNP was degraded, and it could no longer be detected after 24 h in the degradation system (Figure 4C).

The change in concentration of HQ was similar to that of PNP. The HQ concentration steadily increased, peaked at 8 h, and then decreased. Approximately 82.2% of HQ was degraded after 24 h, and it was completely degraded after 3 days (Figure 4D).

β-Ketoadipate, the final hydrolysis product of the transformants BL-MP, was also detected using GC-MS, but its content was very low (data not shown), likely because the degradation of MP by the transformants BL-MP is the result of the dynamic balance between the synthesis and degradation of intermediates. In this balance, all of the intermediates of MP are constantly produced and continuously degraded at the same time, which causes the accumulation of various metabolites to decrease gradually. For example, when the initial concentration of MP was 1 mM, the concentration of PNP peaked at 0.7 mM, and that of HQ peaked at 0.64 mM. Furthermore, β-ketoadipate, the final product of construction, partially entered the TCA cycle, so the accumulation of detected β-ketoadipate was very low.

In BL-control strain, the content of MP was detected over the same period (e.g., 3 days), and no degradation was found (data not shown). The MP hydrolysis products, PNP, HQ, and β-ketoadipate, were also not detected (Figure 4).

Studies on the MP degradation by different microorganisms have been reported. Previously, a *Pseudomonas* sp. strain that could co-metabolically degrade MP was isolated (Chaudhry et al., 1988). Thereafter, *Flavobacterium balustinum*, which is able to utilize MP as the sole carbon source, was reported (Somara and Siddavattam, 1995). Later, *Plesiomonas* sp. strain M6 (Cui et al., 2001) and *Serratia* sp. strain DS001 (Pakala et al., 2007) were also reported to be able to transform MP to *p*-nitrophenol and dimethyl phosphorothioate by hydrolysis; however, further *p*-nitrophenol degradation was not observed. Recently, developments in metabolic engineering have progressed rapidly. *P. putida* KT2440 strain and *P. putida* X3 strain were created by Gong et al. (2016) and Zhang et al. (2016), respectively, to mineralize MP by introducing an MP hydrolase gene (*mpd*) to promote the hydrolysis reaction. These strains can completely mineralize 100 mg/L (approximately 0.38 mM) of MP within 12 and 6 h. Furthermore, strain X3 can also hydrolyze MP to PNP. However, further degradation was not observed (Zhang et al., 2016).

### Analysis of 4-Nitrophenol Hydrolysis

**FIGURE 5:**
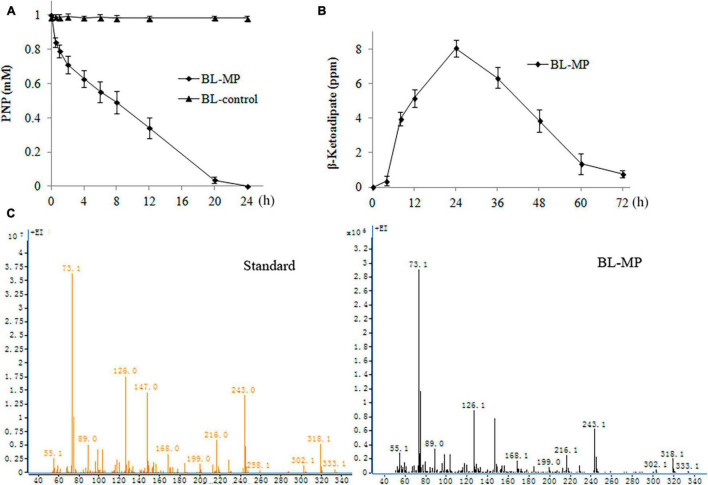
Degradation of 1 mM of 4-nitrophenol (PNP). PNP was converted to β-ketoadipate in the end. Values are mean ± SD of three replicates. **(A)** PNP concentration at different times. **(B)** β-Ketoadipate concentration at different times. **(C)** Mass spectrometry analysis of β-ketoadipate in BL-MP.

PNP, which is widely used in the production of herbicides, pesticides, dye, etc., has been rated as a priority pollutant by US Environmental Protection Agency (EPA) (US Environmental Protection Agency [USEPA], 1976). The PNP biodegradation capability of the transformed strain was detected by the addition of 1 mM of PNP to the prepared bacterial suspension containing inducers. The transformants BL-MP could degrade 50% of PNP within 8 h and 100% of the product within 24 h (Figure 5A).

β-Ketoadipate was also detected. When 1 mM of PNP was added to the liquid medium, the concentration of β-ketoadipate was increased slowly within the first 4 h. The concentration of this product then increased rapidly within 24 h and peaked at 1 day. Thereafter, the concentration of β-ketoadipate gradually decreased (Figures 5B,C). The result illustrates that the β-ketoadipate generated from PNP degradation is quickly consumed to enter the metabolites of multiple anabolic pathways.

In BL-control strain, the content of PNP was detected, and no degradation was found (Figure 5A). The hydrolysis product β-ketoadipate was also not detected (data not shown).

The widespread use of pesticides has introduced organophosphate compounds to the environment through a direct transfer, such as by spraying on agricultural land, and indirect approaches, such as through effluents from various industries and waste materials (Bhatt et al., 2021a). The accumulation of these pollutants may lead to their entry into the food chain, which can result in health hazards to higher animals and humans (Bilal et al., 2019). Thus, the biological breakdown of pesticides, an inexpensive and environment-friendly approach, has become a research hotspot for the detoxification of contaminated sites (Ren et al., 2018). Three phases of microbial remediation have been identified: (1) use of indigenous microbial strains to remove pollutants; (2) application of more advanced techniques, e.g., recombinant DNA technology; and (3) combination of different approaches of systems biology, metabolic engineering, and synthetic biology to understand and reprogram biological systems (Dvořák et al., 2017). Engineered “superbugs” provide an alternative, feasible, and eco-friendly technology for the complete mineralization of pollutants (Ramos et al., 2011). In this study, *E. coli* strain BL-MP was engineered for the remediation of MP via the introduction of six genes, one (*opd*) for MP hydrolysis and the other five (*pnpA*–*pnpE*) for PNP degradation. The recombinant strain could completely degrade 1 mM of MP and 1 mM of PNP within 2 and 24 h, respectively. The β-ketoadipate generated from PNP degradation was quickly consumed to enter the metabolites of multiple anabolic pathways. These results illustrate that the engineered strain BL-MP of *E. coli* could degrade MP efficiently and completely as designed. Future research is required to assess the viability of this system in environmental settings (e.g., soil or plant detritus) and to confirm that such strains can biodegrade MP *in situ*. With these studies as a basis, engineering organisms should degrade other PNP-based organophosphates (parathion and paraoxon) more easily and faster. This study demonstrates the potential of using metabolically engineered organisms in remediation processes for organophosphate pesticides.

## Conclusion

Considering the effects of the extensive use of organophosphate compounds and their toxicity to the environment and biological living systems, the degradation of organophosphate pesticides has attracted considerable attention. Biotic mediators, especially bacteria, which are environmentally friendly and inexpensive, show some ability to degrade toxic organophosphate pesticides into less-toxic byproducts. However, the subsequent biodegradants and their impact on the surrounding environment have not been thoroughly investigated. This work confirms that organophosphate bioremediation may be achieved by using engineered microorganisms. Here, *E. coli* was bioengineered for MP degradation via the introduction of six synthetic genes, namely, *opdS*, *pnpAS*, *pnpBS*, *pnpCS*, *pnpDS*, and *pnpES*, resulting in a new type of transformants, BL-MP. MP and its subsequent decomposition intermediates were completely degraded by these transformants to enter the metabolites of multiple anabolic pathways. The related technologies may be applied to construct the complete bioremediation pathways of MP and potential toxic intermediates. The MP-degrading strain created in this study may be a promising candidate for bioremediation of MP.

## Data Availability Statement

The original contributions presented in the study are included in the article/Supplementary Material, further inquiries can be directed to the corresponding author/s.

## Author Contributions

Q-HY and R-HP designed the research. JX, M-QW, Z-JL, and Y-ST performed these experiments. BW and J-JG analyzed these data. JX wrote the manuscript. All authors contributed to the article and approved the submitted version.

## Conflict of Interest

The authors declare that the research was conducted in the absence of any commercial or financial relationships that could be construed as a potential conflict of interest.

## Publisher’s Note

All claims expressed in this article are solely those of the authors and do not necessarily represent those of their affiliated organizations, or those of the publisher, the editors and the reviewers. Any product that may be evaluated in this article, or claim that may be made by its manufacturer, is not guaranteed or endorsed by the publisher.

## Footnotes

1
http://mall.molbase.cn/

2
http://www.aladdin-e.com/zh_cn/

3
https://www.genscript.com/tools/rare-codon-analysis
