# Supplementary material for: Metabolic Engineering of Escherichia coli for Methyl Parathion Degradation
Source: Front Microbiol. 2022 Feb 11;13:679126. doi: 10.3389/fmicb.2022.679126 (PMC8874220; doi:10.3389/fmicb.2022.679126)
Supplement: Supplementary file 1 [file Table_1.DOCX]

Supporting Information for

Metabolic engineering of *Escherichia coli* for methyl parathion degradation

Jing Xu, Bo Wang, Ming-Qing Wang, Jian-Jie Gao, Zhen-Jun Li, Yong-Sheng Tian*, Ri-He Peng*, Quan-HongYao*

Shanghai Key Laboratory of Agricultural Genetics and Breeding, Biotechnology Research Institute of Shanghai Academy of Agricultural Sciences, Shanghai 201106, China

Jing Xu and Bo Wang are contributed equally to the article.

Table 1: The sequences of primers for respective gene used in this study

| Name | Forward Primer | Reverse Primer |
| --- | --- | --- |
| 16S  OpdS | 5´-ACTCCTACGGGAGGCAGCAG-3´  5´-CCTTACTCTGCCATTGGTCTTG-3´ | 5´-ATTACCGCGGCTGCTGG-3´  5´-GACTCTAAGAGGAACGAAAGC-3´ |
| PnpAS | 5´-CAACTGTGCGTGGTAAGATC-3´ | 5´-CGACCACGACGGAACTCAG-3´ |
| PnpBS | 5´-CAGATGCGTAACTTCCTTG-3´ | 5´-CAGGACCAGCAAGAGTTG-3´ |
| PnpCS | 5´-TGGTCGTTACCGTTCTGAC-3´ | 5´-GTCTTCGTACTTCACTTGC-3´ |
| PnpDS | 5´-TGGGTCCACTGACTTCTG-3´ | 5´-GTCCACAGACCAGAACCCA-3´ |
| PnpES | 5´-CGTGGATGACCCTGAACA-3´ | 5´-GGGATACCAAGTTTCTCG-3´ |
